# Stem cell therapy-based approaches in experimental endometriosis: a systematic review

**DOI:** 10.1186/s12958-026-01553-w

**Published:** 2026-04-17

**Authors:** Isabel Suárez-Carrasco, María Ángeles De Pedro, María Pulido, Verónica Álvarez, Francisco Miguel Sánchez-Margallo, Esther López

**Affiliations:** 1 Stem Cell Therapy Unit, Jesús Usón Minimally Invasive Surgery Centre, Cáceres, Spain; 2 RICORS-TERAV Network, Institute of Health Carlos III, Madrid, Spain; 3 Institute of Molecular Pathology Biomarkers, University of Extremadura, Cáceres, Spain

**Keywords:** Endometriosis, Stem cells, Preclinical models

## Abstract

**Background:**

Stem cell-based therapies have emerged as a promising approach for endometriosis due to their regenerative and immunomodulatory properties. Although numerous preclinical studies have reported beneficial effects, no systematic synthesis has yet evaluated their impact, the models employed and associated biomarkers. Therefore, the aim of this systematic review is to synthesise all available preclinical evidence (both in vitro and in vivo) assessing stem cell-based therapies and stem cell-derived products in experimental models of endometriosis.

**Methods:**

Following PRISMA guidelines, PubMed, Scopus and Web of Science were searched from 1 January 2015 up to 11 February 2025. Preclinical studies (in vitro and in vivo) evaluating stem cell-based therapies in experimental models of endometriosis were included. Data were extracted regarding the models employed, cell sources, analysed biomarkers and reported cellular outcomes.

**Results:**

Twenty preclinical studies were included, employing a wide range of in vitro models (most commonly endometriotic cells) and predominantly murine in vivo models induced by intraperitoneal injection of uterine tissue. Adipose-derived stem cells (ADSCs) and bone marrow-mesenchymal stem cells (BMMSCs) were the most frequently investigated cell sources. Across the studies, *VEGFA, TNFA*, metalloproteinases (*MMPs*) and components of the PI3K/AKT/mTOR pathway were the most consistently analysed biomarkers, primarily assessed by PCR and immunohistochemistry. Overall, 72 of the 92 reported outcomes were positive, with ADSCs demonstrating the most pronounced therapeutic effects and being characterised by their immunomodulatory, anti-inflammatory and regenerative activity. Negative or neutral findings, such as increased cellular adhesion and invasion, were limited and largely associated with ADSCs and umbilical cord mesenchymal stem cells (UCMSCs).

**Conclusions:**

Stem cell-based therapies demonstrate promising effects in experimental endometriosis, with most studies reporting improvements in lesion characteristics and inflammatory pathways. However, substantial variability in models, cell types and outcomes limits the strength of current evidence. More standardised and rigorous preclinical research is needed to confirm these findings and support the translation of stem cell therapy-based approaches into future clinical practice.

**Supplementary Information:**

The online version contains supplementary material available at 10.1186/s12958-026-01553-w.

## Introduction

Endometriosis is an inflammatory-based condition that affects approximately 10% of women of reproductive age worldwide [1], although its actual prevalence may be higher, as it is often underdiagnosed [2]. This disease is characterised by the presence of endometrial-like tissue outside the uterus, frequently causing chronic pain, dysmenorrhoea and even infertility [3]. Moreover, endometriosis has been associated with an increased risk of developing other conditions such as ovarian cancer, cardiovascular disease, autoimmune disorders and mental health issues [4]. The heterogeneity of its phenotypes, its unknown pathogenesis and its frequent comorbidity with other conditions make endometriosis particularly challenging to diagnose [5]. Among all available techniques, laparoscopy is considered the gold standard for diagnosing endometriosis, as it allows both direct visualisation of the lesions and the collection of biopsies for histopathological confirmation [6]. However, its role as a diagnostic tool has been increasingly questioned in recent years because it is unable to detect early-stage disease [7]. As a result, not all patients receive a timely diagnosis, remaining exposed to unnecessary surgical risks and associated costs [6]. All of this, combined with the lack of specific biomarkers that can accurately diagnose endometriosis, underscores the urgent need to identify new biological markers. These could enable the development of non-invasive diagnostic tests that improve patients’ quality of life and support earlier diagnosis and treatment [2], especially considering that the estimated delay from the onset of symptoms to diagnosis ranges from 7 to 10 years [8].

The medical treatment of endometriosis primarily focuses on pain relief through non-steroidal anti-inflammatory drugs (NSAIDs) and hormonal therapies such as combined oral contraceptives, progestins and gonadotrophin-releasing hormone (GnRH) agonists or antagonists [9]. While these approaches can alleviate some symptoms and improve quality of life, they do not prevent recurrence or resolve the disease. Moreover, a subset of patients shows limited response, likely due to the heterogeneous distribution of oestrogen and progesterone receptors in lesions [10]. These therapeutic limitations highlight the pressing need for novel strategies.

In this context, stem cell-based therapies have emerged as a promising alternative, in the setting of inflammatory and immune-mediated diseases, due to their well-established capacity to modulate innate and adaptive immune responses [11]. In particular, mesenchymal stem/stromal cells (MSCs) have shown therapeutic potential in conditions such as graft-versus-host disease, inflammatory bowel disease, rheumatoid arthritis, multiple sclerosis and systemic lupus erythematosus, where their immunosuppressive and anti-inflammatory properties contribute to disease amelioration [11]. This immunomodulatory profile is highly relevant to endometriosis, a chronic inflammatory disease characterised by immune dysregulation, persistent inflammation, aberrant angiogenesis, fibrosis and resistance to apoptosis. Accordingly, stem cell-based therapies have been proposed as a strategy to counteract key pathological processes underlying endometriosis, warranting investigation of their effects first at the preclinical level before considering clinical application.

Preclinical studies, including both in vitro and in vivo approaches, are a fundamental step in the development of new therapeutic strategies. They provide essential insights into efficacy, mechanisms of action and potential safety concerns before clinical translation. In the context of stem cell-based therapies for endometriosis, these models allow researchers to evaluate how therapeutic stem cells interact with endometriotic lesions, modulate inflammatory responses and influence disease progression. In vitro studies offer a controlled environment to analyse specific cellular behaviours and molecular pathways, while i*n vivo* models replicate the complex physiological and pathological conditions of the disease [12].

Together, these preclinical systems are indispensable for guiding the design of future clinical applications and ensuring a solid scientific foundation. Given this context, there remains a clear need to comprehensively evaluate the evidence generated to date. Accordingly, the main objective of this systematic review is to synthesise all available preclinical evidence (both in vitro and in vivo) that evaluates stem cell-based therapies and stem cell-derived products in experimental models of endometriosis. Therefore, conducting a systematic review to synthesise and critically analyse all available preclinical evidence is crucial to identify the most suitable preclinical models, the most appropriate biomarkers for diagnosis and the potential of stem cell-based therapies. This approach will clarify the current state of knowledge and identify methodological limitations and research gaps that require further investigation. In doing so, it will provide a robust framework for the rational design of future translational and clinical studies aimed at improving the management of endometriosis.

## Methods

### Search strategy

According to the guidelines of the Preferred Reporting Items for Systematic Reviews and Meta-Analyses (PRISMA), a structured search was conducted in the databases PubMed, Scopus and Web of Science. For this purpose, terms related to the application of stem cell-based therapies in preclinical models of endometriosis were used, resulting in the following search string: ("endometriosis") AND ("model*" OR "in vitro" OR "in vivo") AND ("stem cell*" OR "conditio* medi*" OR "exosom*" OR "mesenchym* stem cell*" OR "paracri* fact*" OR "paracri* signal*" OR "vesicl*"). Only studies published within the last ten years (between 1 January 2015 and 11 February 2025) were included. This review was registered with PROSPERO on 24 June 2025 under the registration number CRD420251071106.

### Inclusion and exclusion criteria

Preclinical studies, both in vitro and in vivo, written in English and in which stem cells or their products were administered therapeutically to an endometriosis model were included. Studies that were not original studies were discarded, so other reviews, conference abstracts, letters to the editor, etc. were discarded. In silico studies, studies not involving endometriosis, clinical phase studies and studies in which other therapies besides stem cell-based therapies were administered were also excluded.

### Data extraction and quality assessment

After searching in the three aforementioned databases, the records obtained from each of them were exported to the Parsifal platform to facilitate data extraction. To discard studies that did not meet the established requirements, the following order of priority was established among the exclusion criteria: 1) not written in English, 2) other reviews, 3) incomplete studies, 4) in silico studies, 5) studies not involving endometriosis, 6) studies conducted in humans, 7) studies applying therapies other than stem cell-based therapies or not applying any therapy and 8) full text not available.

The risk of bias assessment of the studies was conducted using the Parsifal platform. For the in vivo studies, the SYRCLE risk of bias tool [13] was used to generate a set of questions and establish a scoring system: 2 points were awarded if the article fully met the criterion, 1 point if it partially met the criterion and 0 points if it did not meet the criterion. Each article could achieve a maximum score of 44 points. For the in vitro studies, although no standardised tool is currently available, the QUIN tool was applied [14] using the same scoring system described above for the in vivo studies. Articles scoring 26 points or fewer (≤ 60% of the maximum score) were considered very high-risk items and were excluded from the study [15]. Only studies scoring above 26 were selected for final data extraction and subjected to a more detailed bias analysis, in which five potential sources of bias were examined. Each individual source of bias was classified as “low risk”, “high risk”, or “moderate risk”, resulting in an overall risk of bias judgement for each article in accordance with the Cochrane Handbook guidelines [16]. Finally, the robvis tool was used to generate the risk of bias figure (Supplementary Fig. 1).

After data extraction, the in vitro articles were classified according to twelve different categories: 1) endometriosis model employed, 2) type of cell used as a model, 3) culture system, 4) techniques used to characterise the model, 5) biomarkers analysed, 6) techniques used to study biomarkers, 7) stem cell type or stem cell-derived products administered, 8) type of therapy administered, 9) species from which the stem cells derived, 10) therapeutic transplantation type, 11) effects observed post-treatment and 12) type of observed effect (Supplementary Table 1). For in vivo studies, the following categories were also added: 13) immune status of the model, 14) method of model induction, 15) species of origin of the tissue used to induce the model, 16) model transplantation type, 17) whether the biomarker was measured locally or systemically and 18) route of administration (Supplementary Table 2).

The effects observed post-treatment were subsequently classified as positive, negative, or neutral based on their biological impact relative to control conditions. Positive effects were defined as outcomes indicative of a therapeutic benefit, including reduced proliferation, invasion, inflammation, angiogenesis, fibrosis, or lesion size, as well as increased apoptosis or improved fertility-related parameters. Negative effects were defined as outcomes suggestive of disease progression or exacerbation, such as increased proliferation, invasion, angiogenesis, adhesion, or inflammatory responses. Neutral effects corresponded to the absence of significant or measurable changes compared with control groups. A detailed classification of all reported effects and their corresponding categories is provided in Supplementary Tables 1 and 2.

The entire process was carried out by two independent reviewers, with discrepancies being resolved by consensus between the two reviewers. In case of doubt, the article passed to the next phase of analysis.

### Data management

Following completion of the article selection process, the extracted data were compiled in Microsoft Excel to facilitate organisation and analysis and subsequently arranged into tables according to the predefined categories. Statistical processing and data visualisation were performed using GraphPad Prism (version 9.5.1) and RAWGraphs2.0.

## Results

### Article selection and quality assessment

The search identified 905 records: PubMed (*n* = 213), Web of Science (*n* = 335) and Scopus (*n* = 357). After removal of duplicates (*n* = 371), 534 records remained. Manual screening by title and abstract excluded 425 records.

A total of 109 studies underwent full-text review. Of these, 86 were excluded by: other therapies (*n* = 75), disease other than endometriosis (*n* = 7), not in English (*n* = 1), human studies (*n* = 1), incomplete article (*n* = 1) and inaccessible (*n* = 1).

**Fig. 1:**
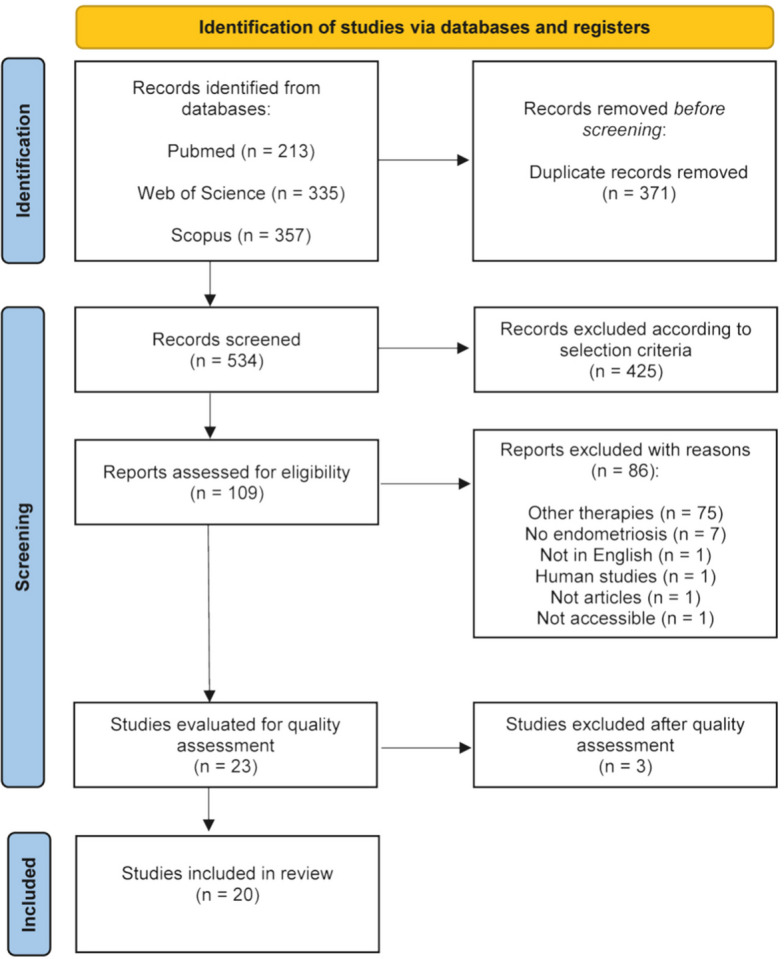
PRISMA flow diagram of the study selection process, including identification, screening and final inclusion. The literature search was conducted on 11 February 2025

Twenty-three studies were assessed for quality. Three were excluded [17–19] for scoring ≤ 26 points in the risk of bias assessment. Twenty studies were included in the final data extraction (Fig. 1).

The studies included in the review were found to have a high or moderate risk of bias, particularly in domains related to random allocation of study subjects and the implementation of blinding for outcome assessment (Supplementary Fig. 1).

### Preclinical models of endometriosis

In this review, preclinical models of endometriosis were analysed using in vitro and in vivo approaches. Of the 20 original studies included, 7 exclusively performed in vitro experiments, 11 used animal models and 2 included both approaches.

**Fig. 2:**
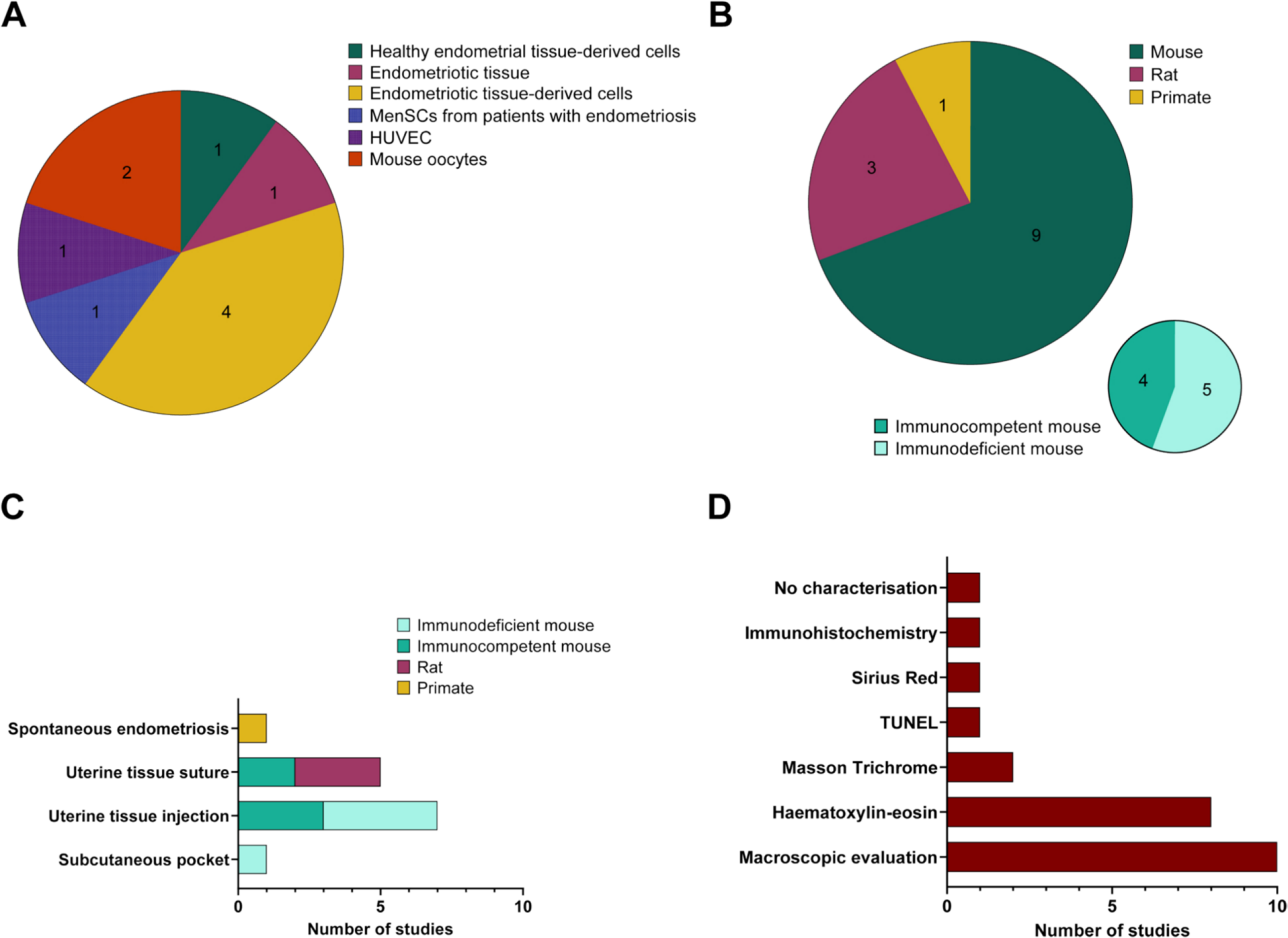
Preclinical models of endometriosis used in the reviewed studies. **A** Pie charts illustrating the number of times the different types of in vitro models have been used and **B** the number of times each animal model has been employed across the in vivo studies, additionally indicating the number of times immunocompetent and immunodeficient mouse models were used. **C** Bar charts showing the number of studies in which the different methods of induction and **D** characterisation of the in vivo models were used. HUVEC: Human umbilical vein endothelial cells; MenSCs: Menstrual blood-derived mesenchymal stem cells; TUNEL: Terminal deoxynucleotidyl transferase-mediated dUTP nick end labelling

In the in vitro studies, various cell types were used as experimental models, including healthy endometrial tissue-derived cells, endometriotic tissue, endometriotic tissue-derived cells, mouse oocytes, human umbilical vein endothelial cells (HUVEC) and menstrual blood-derived mesenchymal stem cells (MenSCs) from patients with endometriosis. All these experiments were conducted using two-dimensional (2D) culture systems, except for the only study that used a tissue-based model [20]. The results showed that endometriotic tissue-derived cells are the most studied, being used 4 times in the in vitro studies analysed (Fig. 2A). One of these studies [21] used endometriotic tissue-derived cells obtained from two different sources, cysts and endometrial tissue from patients with endometriosis, which were considered as two distinct cell lines within the same study.

Regarding in vivo studies, animal models included primates, rats and mice, with the latter being employed in 9 of the in vivo studies. Furthermore, analysis of the immunological status of the animal models prior to endometriosis induction revealed that only mice were used under immunodeficient status, being used in 5 of the 9 studies in which mice were employed (Fig. 2B).

Three different induction methods were identified in mouse models: 1) intraperitoneal or subcutaneous injection of uterine tissue, 2) implantation of uterine tissue by suture and 3) creation of a subcutaneous pocket followed by implantation of ectopic uterine tissue. Among these, injection of uterine tissue was the predominant approach, applied in 70% of mouse studies and 50% of all in vivo studies. In rat models, only the implantation of uterine tissue by suture was used. In the primate model, animals with spontaneous endometriosis were studied. Interestingly, immunodeficient mice were induced using uterine tissue injection and subcutaneous pocket methods, employing allogeneic human tissue (Fig. 2C).

Characterisation of endometriosis models involves confirming the successful establishment of the pathology to ensure that therapeutic effects are evaluated in a validated disease context. In most reviewed studies, this assessment was performed prior to treatment initiation to confirm model induction. However, in some in vivo models where the disease is consistently established, characterisation was conducted at the end of the study, particularly in vehicle-treated animals, to confirm the presence of the pathology and to provide a valid baseline for comparison with the treated groups. In the case of in vitro studies, only the article using a tissue-based model [20] characterised its model by haematoxylin–eosin staining. Several approaches were used for this purpose in in vivo studies, including macroscopic evaluation, histological staining and immunohistochemical analysis. Macroscopic evaluation, reported in 64% of the studies, was the most frequently used method and involved measuring cyst size based on weight and volume, as well as assessing lesion number and gross appearance. Histological analyses included haematoxylin–eosin, Masson’s trichrome, TUNEL and Sirius Red staining, with haematoxylin–eosin staining being the second most commonly employed technique (50% of the studies), mainly to confirm the presence of endometrial glandular and stromal tissue within the lesions. Notably, one of the reviewed studies [22] did not report any form of model characterisation (Fig. 2D).

### Stem cell therapy administrations

The systematic review identified several types of stem cells that have been investigated as potential therapies for endometriosis in preclinical models. Table 1 summarises the frequency of use for each cell type, distinguishing between in vitro and in vivo studies.

**Table 1:** Types of stem cells administered

Administered cell type	N.S.*	Total%	Type of study	Type of study%	References
Adipose-derived stem cells	7	32	In vivo	14	[23–25]
			In vitro	18	[20, 21, 26, 27]
Bone marrow mesenchymal stem cells	6	27	In vivo	18	[22, 28–30]
			In vitro	9	[22, 31]
Endometrial mesenchymal stem cells	4	18	In vivo	14	[32–34]
			In vitro	5	[34]
Umbilical cord mesenchymal stem cells	3	14	In vivo	9	[35, 36]
			In vitro	5	[37]
Wharton’s jelly stem cells	1	5	In vitro	5	[38]
Menstrual blood-derived mesenchymal stem cells	1	5	In vitro	5	[39]

^*^N.S.: number of studies

Adipose-derived stem cells (ADSCs) were the most frequently studied cell type in both in vitro and in vivo studies, being administered in approximately 32% of the studies included in this review. The next most commonly used cell type was bone marrow mesenchymal stem cells (BMMSCs); however, their use in in vitro studies was half as frequent. Other cell types including endometrial mesenchymal stem cells (eMSCs) and umbilical cord mesenchymal stem cells (UCMSCs) were studied less frequently. Lastly, Wharton's jelly stem cells (WJSCs) and MenSCs were each administered in only one in vitro study (Table 1). Additional data showed that all stem cell-based treatments were allogeneic in the in vitro studies, whereas in the in vivo studies the distribution of allogeneic and xenogeneic stem cell treatments was more balanced (Supplementary Tables 1 and 2).

**Fig. 3:**
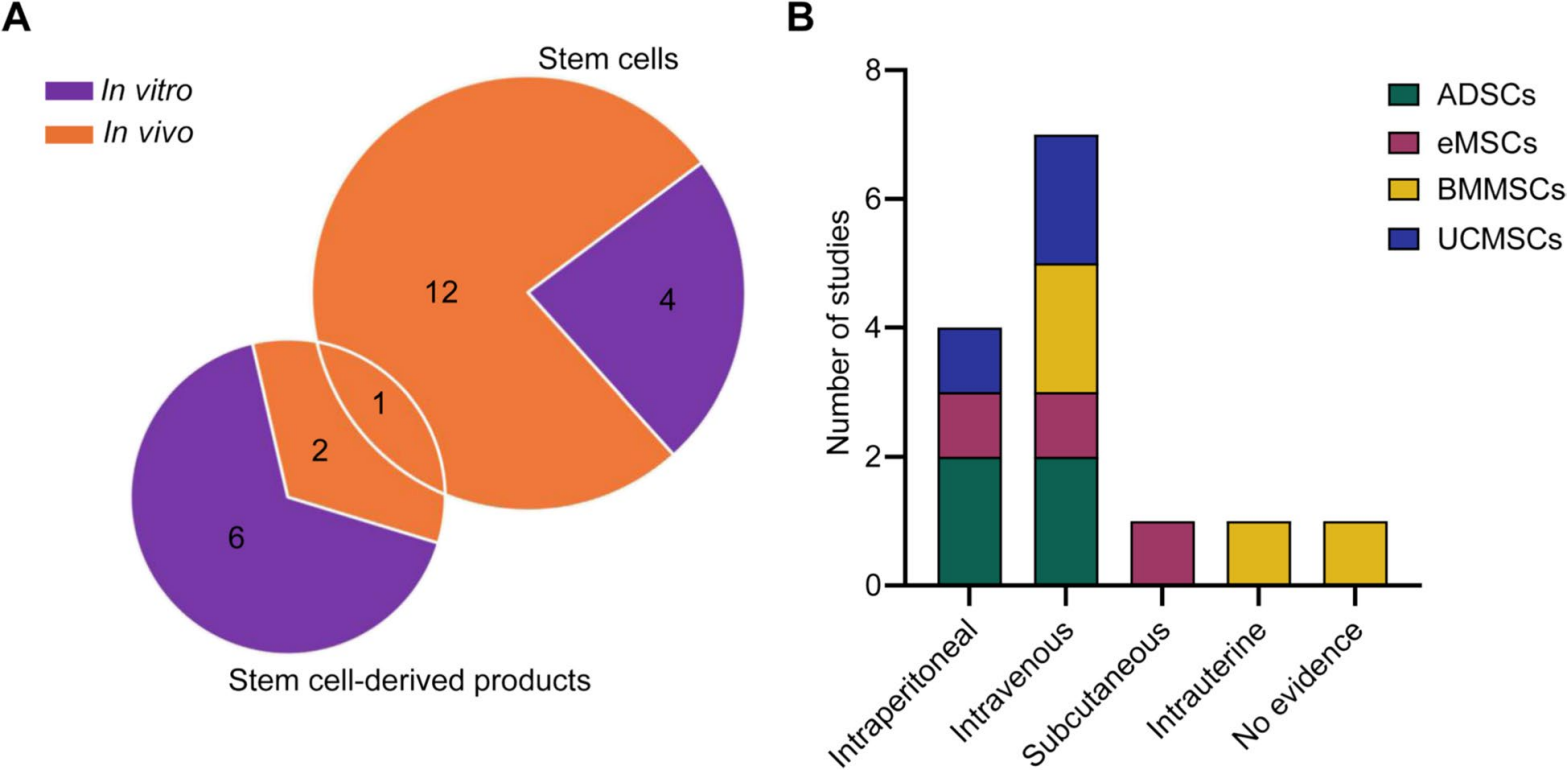
Therapeutic approaches and administration routes of cell-based therapies in experimental endometriosis. **A** Distribution of studies applying cells directly versus cell-derived products based on in vitro (purple) and in vivo (orange) experimental settings. **B** Administration routes of various cell types in in vivo studies. The bar chart shows the frequency of different types of administration. ADSCs: Adipose-derived stem cells; BMMSCs: Bone marrow mesenchymal stem cells; eMSCs: Endometrial mesenchymal stem cells; UCMSCs: Umbilical cord mesenchymal stem cells

Among studies using only stem cells for treatments, 12 were in vivo and 4 were in vitro. By contrast, stem cell-derived products (such as cell lysates, conditioned media, extracellular vesicles, or exosomes) predominated in vitro, being used in 6 of the studies, while only 2 of the in vivo studies employed this approach. Notably, only one in vivo study administered a combination of ADSCs and their products, specifically conditioned medium (Fig. 3A). The routes of administration were also analysed across the in vivo studies, identifying four distinct approaches: intravenous, intraperitoneal, subcutaneous and intrauterine. The intravenous route was the most frequently used, followed by intraperitoneal administration. Subcutaneous and intrauterine routes were less common, each reported in only one study, while one study [29] did not specify the administration route (Fig. 3B).

### Biomarkers

**Fig. 4:**
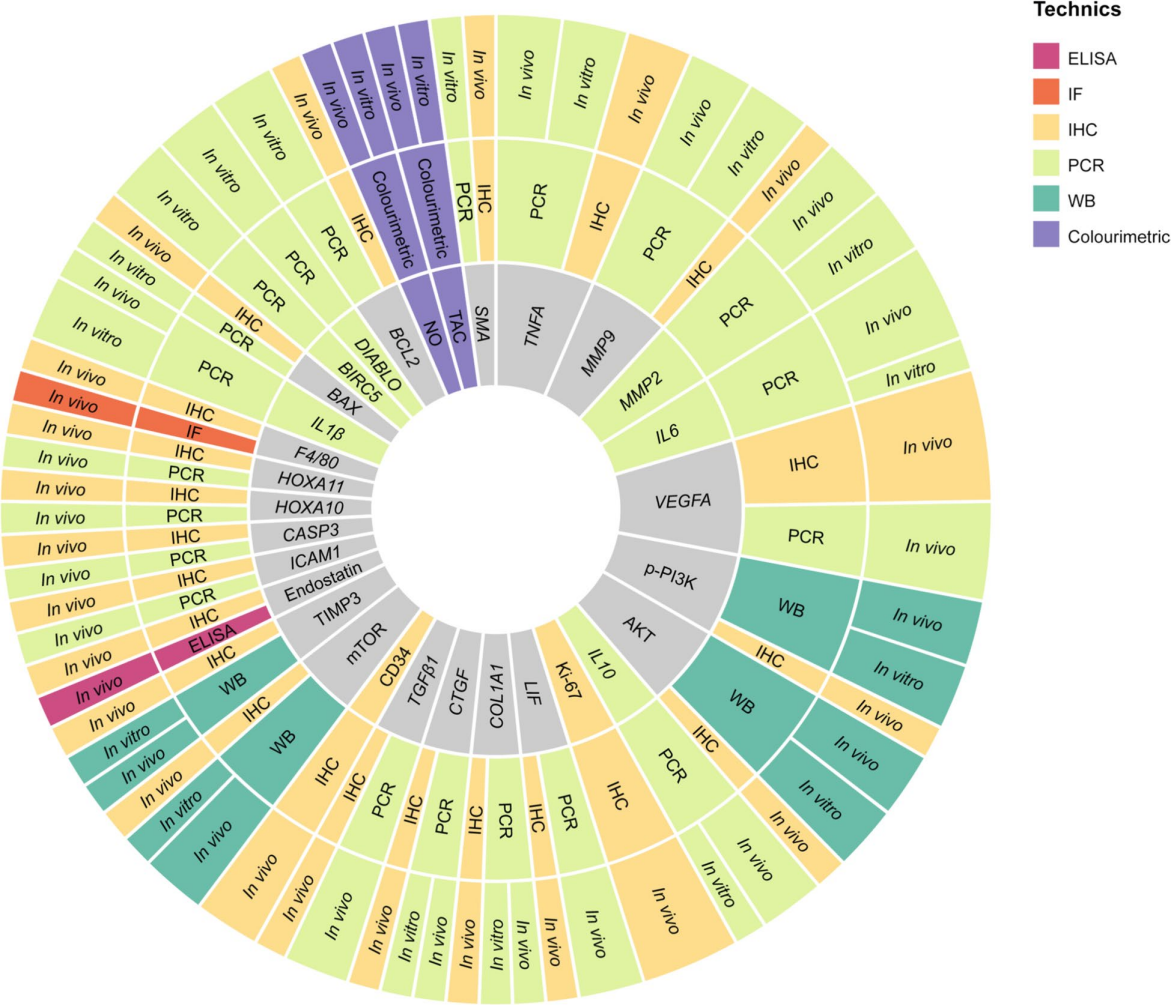
Sunburst diagrams illustrating biomarker analysis according to the techniques used in the selected in vitro and in vivo studies. Representation of biomarkers analysed, categorised by the detection technique employed and their type of study. Each colour within the outer segments corresponds to a distinct technique, as detailed in the legend. Biomarkers shown in grey within the inner circle indicate those assessed by more than one technique. The size of each section is proportional to the number of times each biomarker was analysed in the different studies included. Biomarkers were named in accordance with the standardised human gene symbol when both gene and protein expression, or gene expression alone, were determined; when assessed solely at the protein level, the human protein nomenclature was used. Only those biomarkers that were detected at least twice in the studies included in this review were selected for this part of the analysis. Colourimetric: colourimetric assays; ELISA: Enzyme-Linked Immunosorbent Assay; IF: Immunofluorescence; IHC: Immunohistochemistry; PCR: Polymerase Chain Reaction; WB: Western blotting; TAC: Total antioxidant capacity; NO: Nitric oxide

Based on our selection criteria, only biomarkers reported in at least two independent studies were included in the analysis. The combined in vitro and in vivo assessment identified a total of 30 different biomarkers, which were evaluated using six main technical approaches: Immunohistochemistry (IHC), Polymerase Chain Reaction (PCR), Western blotting (WB), Enzyme-Linked Immunosorbent Assay (ELISA), immunofluorescence (IF) and other colourimetric assays. Biomarkers were named in accordance with the standardised human gene symbol in cases where only gene expression was determined; when assessed solely at the protein level, human protein nomenclature was applied. In cases where the same biomarker was evaluated at both gene and protein expression levels, the human gene nomenclature was used. Among these, *VEGFA, TNFA, p-PI3K, MMP9, AKT, MMP2, IL6* and mTOR were the most frequently investigated. IHC and PCR emerged as the two most commonly employed detection techniques (Fig. 4). In the in vivo studies, biomarker assessment was predominantly performed locally in biopsies obtained from endometriotic lesions or ovarian endometriotic cysts, with the exception of endostatin, total antioxidant capacity (TAC) and nitric oxide (NO), which were measured systemically in blood samples (Supplementary Table 1).

**Fig. 5:**
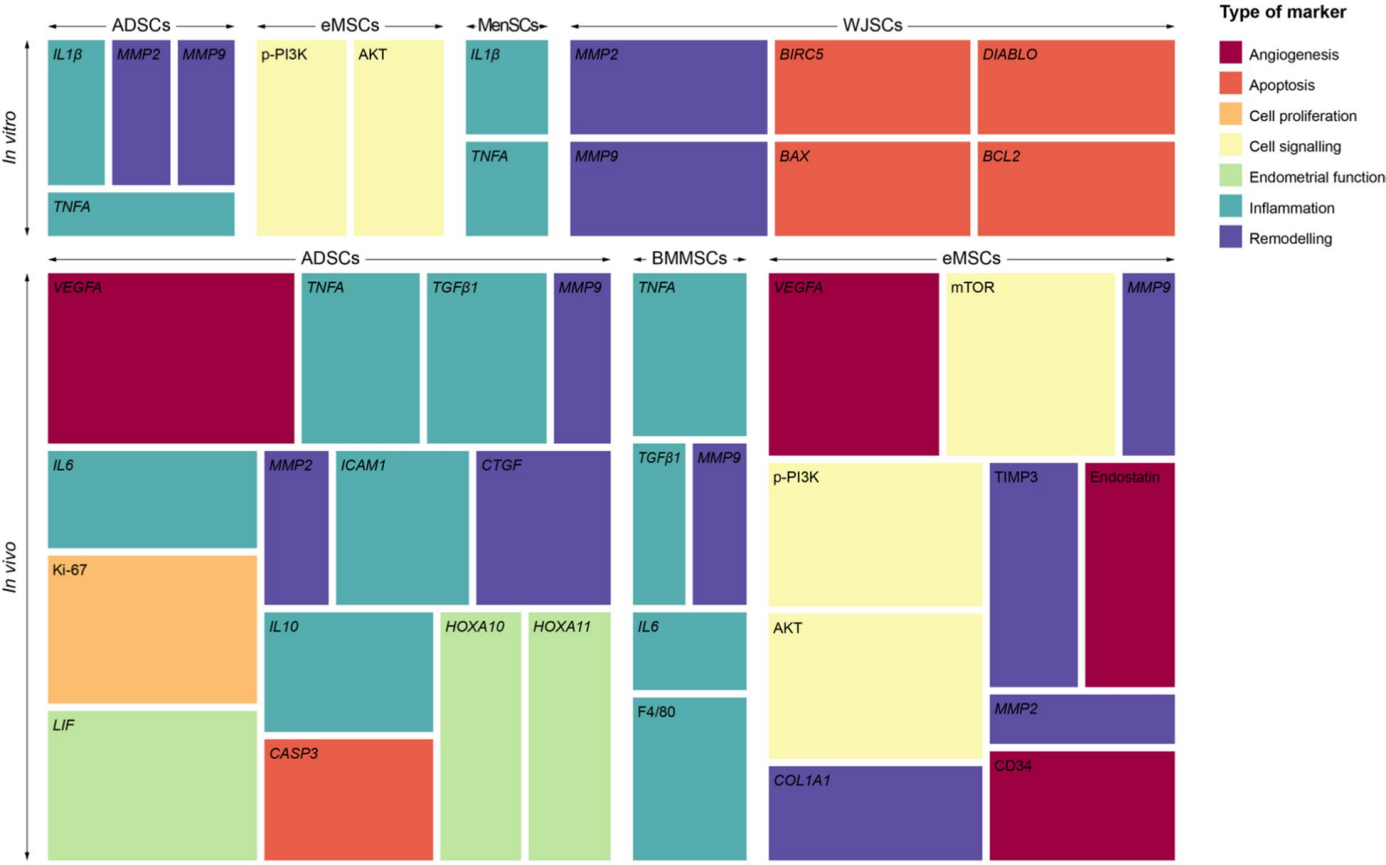
Treemap illustrating the distribution of analysed biomarkers by type of marker, stem cell type and study setting. The upper section of the treemap represents in vitro studies, while the lower section corresponds to in vivo studies. Each block signifies a specific stem cell type, as detailed in the legend. Within these blocks, individual-coloured rectangles represent a specific kind of biomarker, as detailed in the legend. The size of each rectangle is proportional to the relative frequency or number of times that particular biomarker was reported in conjunction with the respective cell type and study setting within the reviewed literature. Only those biomarkers that were detected at least twice in the studies included in this review were selected for this part of the analysis. ADSCs: Adipose-derived stem cells; BMMSCs: Bone marrow mesenchymal stem cells; eMSCs: Endometrial mesenchymal stem cells; MenSCs: Menstrual blood-derived mesenchymal stem cells; WJSCs: Wharton's jelly stem cells

The analysis of previously mentioned biomarkers in relation to the administered stem cell types revealed distinct patterns across in vitro and in vivo studies (Fig. 5). In the in vitro experiments, ADSCs and MenSCs modulated inflammatory and tissue remodelling, showing engagement with a range of inflammatory and remodelling markers, including *IL1β*, *TNFA*, *MMP2* and *MMP9*. WJSCs were associated with markers related to cell survival and apoptosis: *BIRC5*, *BAX* and *DIABLO*. eMSCs influenced cell signalling and proliferation pathways, particularly p-PI3K and AKT.

Conversely, in in vivo studies, ADSCs were associated with a broad spectrum of biomarkers including *VEGFA*, *IL6*, Ki-67 and *CASP3*, indicating their multifaceted impact. BMMSCs were associated with inflammatory markers like *TNFA*, *IL6* and, along with immune cell markers such as F4/80, underscoring their immunomodulatory potential. eMSCs exhibited a prominent role in modulating a diverse set of biomarkers, including mTOR, TIMP*3, MMP9*, endostatin and CD34, reflecting their involvement in matrix remodelling and cell proliferation.

### Cellular effects

**Fig. 6:**
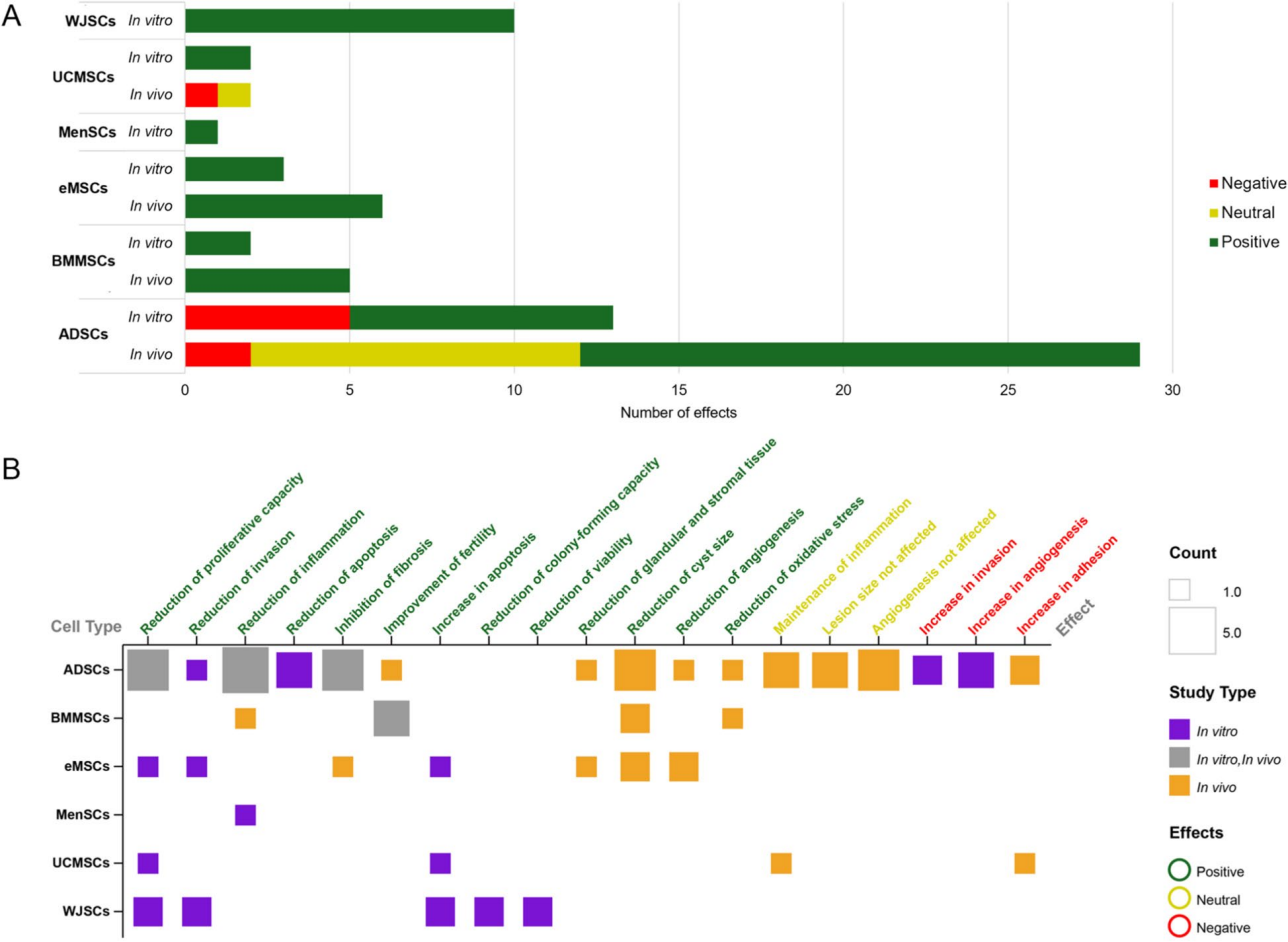
Effects of stem cell-based therapies in experimental endometriosis studies. **A** Bar chart of total number of individual positive (green), negative (red) and neutral (yellow) effects reported per cell type, highlighting the overall contribution of each cell type to the observed therapeutic outcomes and study types (in vitro vs. in vivo). **B** Matrix plot representation detailing specific effects produced by each cell type in in vitro (purple), in vivo (orange), or both (grey) study types. Each square represents a specific effect, with colour indicating the study type and size correlating with the count of times that effect was reported for a given cell type. Positive effects are indicated in green, negative effects in red and neutral effects in yellow. Only 19 effects that were reported at least twice in the studies included in this review were selected for this part of the analysis. ADSCs: Adipose-derived stem cells; BMMSCs: Bone marrow mesenchymal stem cells; eMSCs: Endometrial mesenchymal stem cells; MenSCs: Menstrual blood-derived mesenchymal stem cells; UCMSCs: Umbilical cord mesenchymal stem cells; WJSCs: Wharton's jelly stem cells

Across the 20 studies included in this review, a total of 92 individual effects attributed to the administered therapies (stem cells, stem cell-derived products and combination of both treatments) were reported. ADSCs prominently featured, accounting for 52 effects, representing approximately 56% of all observed outcomes. In contrast, BMMSCs and eMSCs demonstrated a considerably lower number of effects, with 11 detections for each cell type (Fig. 6A).

For the analysis of observed effects, the 92 individual effects mentioned above were subsequently categorised as positive, negative, or neutral. A substantial majority of effects following stem cell therapy were positive (72), with a low number of negative effects (11) and even fewer neutral effects (9). These individual effects were grouped into 31 distinct effects, of which, only 19 effects that were reported at least twice in the selected studies were considered. All cell lines demonstrated some positive effects, with ADSCs proving most effective, particularly in in vivo settings. In in vitro assays, all cell types exhibited positive outcomes, with WJSCs being the most abundant contributors. Negative effects were exclusively observed with ADSCs and UCMSCs, primarily in vivo. Similarly, neutral effects were also associated with ADSCs and UCMSCs, in this instance, solely within in vivo assays (Fig. 6A).

Up to four different cell types (ADSCs, UCMSCs, eMSCs and WJSCs) demonstrated the ability to reduce the proliferation of target cells, predominantly in in vitro studies. Furthermore, effects such as a reduction in invasion, a decrease in inflammation, a reduction in cyst size, or an increase in apoptosis were reported with three of the cell types analysed (Fig. 6B). Upon detailed examination of the specific effects elicited by each cell line, a primary finding for ADSCs was the reduction of inflammation, evident in both in vitro and in vivo contexts. Other significant effects attributed to ADSCs included a reduction in proliferative capacity of endometriotic cells, reduction of cyst size and inhibition of fibrosis.

**Fig. 7:**
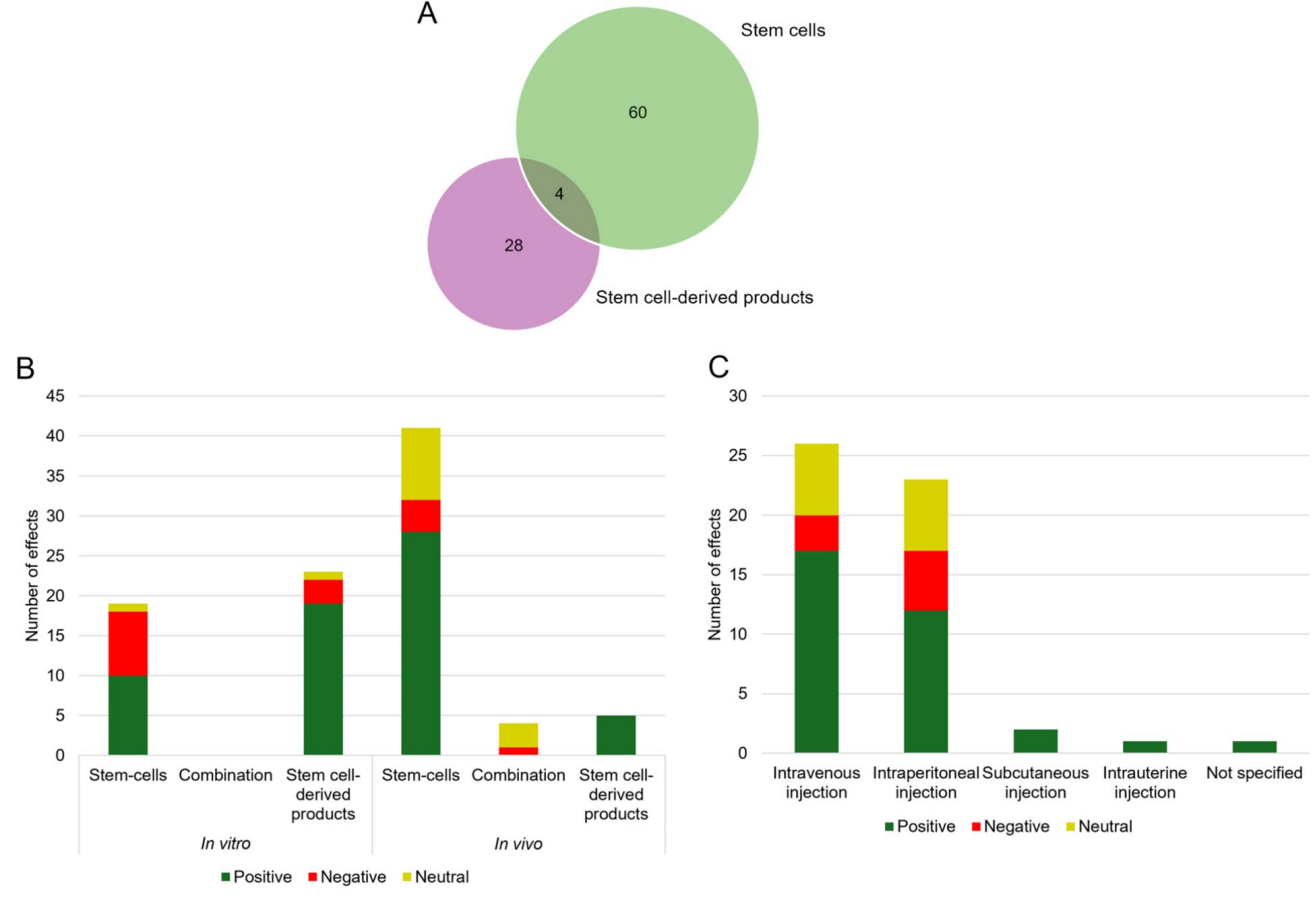
Therapeutic approaches and administration routes in relation to observed effects. **A** The Venn diagram illustrates the overlap and distinct contributions of direct stem cell application (green) and stem cell-derived products therapies (purple) to the total reported effects. **B** Bar charts depict the distribution of positive (green), negative (red) and neutral (yellow) effects across different therapeutic approaches in in vitro and in vivo studies and **C** considering specific administration route employed in in vivo studies

Further analysis investigated the outcomes according to the therapeutic strategy. Of the 92 individual effects mentioned above, a total of 60 effects were attributed only to direct stem cell administration. While 28 effects were exclusively linked to the use of stem cell-derived products, only 4 effects were observed when both cells and their products were combined (Fig. 7A).

When stratifying effects by their nature (positive, negative, neutral) across different study settings, positive outcomes were dominant in both in vitro and in vivo experiments. In in vitro studies, therapies based on stem cell-derived products generated slightly more positive effects (19 positive, 3 negative, 1 neutral) compared with stem cell treatments (10 positive, 8 negative, 1 neutral), with no combination approaches tested. In in vivo studies, direct stem cell administration produced the highest number of positive outcomes (28 positive, 4 negative, 9 neutral), whereas stem cell-derived products exclusively reported positive effects (5). Conversely, combination strategies failed to produce positive effects, with only one negative and three neutral outcomes reported (Fig. 7B).

Regarding the route of administration, intravenous injection accounted for the largest number of positive effects (17), followed by intraperitoneal injection (12), both with similar proportion of negative and neutral effects. Subcutaneous and intrauterine routes were reported in single studies but consistently yielded positive outcomes (Fig. 7C).

## Discussion

It has been demonstrated that stem cell-based therapies and their derived products have promising effects in reducing endometrial fibrosis, promoting repair of damaged endometrium and improving uterine receptivity [40]. These effects have been explored in other gynaecological disorders, including Asherman’s syndrome, endometrial injury, uterine adhesions and uterine prolapse, with several clinical trials currently registered evaluating these therapeutic approaches (NCT02144987, NCT05343572, NCT07176143, NCT06896747). However, no clinical trial has yet been registered applying stem cell–based therapies or their derived products in the case of endometriosis. Together with the limited number of studies published over the past 10 years, this reflects the slow progress in investigating stem cells and their derived products as potential treatments for endometriosis. Therefore, a comprehensive compilation and analysis of the available preclinical data is required, which constitutes the focus of this review and aims to facilitate the translation of preclinical findings into clinical practice.

Preclinical studies play a fundamental role in advancing our understanding of disease mechanisms and in evaluating the safety and efficacy of emerging therapeutic strategies, thereby supporting their successful translation into clinical practice. Within this context, the development and use of robust and physiologically relevant preclinical models of endometriosis are essential. Among in vitro approaches, endometriotic tissue-derived cells isolated directly from patients with endometriosis through surgical procedures were most commonly employed. Moreover, stromal cells were the cell type most frequently used to generate the in vitro models analysed in this review. These cells are often used to assess key disease-related processes, such as angiogenesis and inflammation [41]. However, they present the inherent limitations of in vitro studies, including the progressive loss of phenotypic and genetic characteristics of the tissue of origin after repeated passages, which may compromise the validity of experimental findings [42]. From an in vivo perspective, the best available models for the study of endometriosis remain non-human primate models, owing to their ability to menstruate regularly and to develop endometriosis spontaneously. However, ethical, logistical and economic constraints limit their use [43]. Nonetheless, in this review, there was a clear preference for the use of mouse models, most of which were induced by intraperitoneal injection of uterine tissue, as this is the most effective induction method for reproducing the peritoneal environment of human endometriosis [44]. The use of immunodeficient mouse models was restricted exclusively to studies in which human tissue was used to induce the disease. These immunodeficient murine models prevent graft rejection, thereby promoting implantation and the development of vascularised lesions characteristic of endometriosis [45]. Despite their practical advantages, these models differ substantially from human reproductive physiology, which may limit their translational relevance and contribute to discrepancies between preclinical and clinical outcomes.

Proper characterisation of in vitro and in vivo endometriosis models is essential to ensure their biological relevance. However, none of the in vitro studies included in this review performed such comprehensive validation, with characterisation being limited to histological analysis in a single tissue-based model [20], despite cells isolated from endometriotic lesions being expected to exhibit a defined molecular and functional profile (high ERβ, MMP2 and COX-2 expression, low progesterone receptor expression and secretion of pro-inflammatory cytokines and chemokines) [41]. On the other hand, most in vivo models were primarily assessed through macroscopic evaluation and histological confirmation of lesion presence using haematoxylin–eosin staining, which enables visualisation of glandular and stromal structures and glandular transformation [46], as well as the assessment of other processes such as angiogenesis [47].

Furthermore, biomarkers represent a highly valuable tool for the characterisation of pathological conditions and for assessing the efficacy of a given treatment [48]. Among the biomarkers analysed in the present review, *VEGFA*, *MMPs*, *TNFA* and *IL6* emerge as closely interconnected mediators within the inflammatory and angiogenic microenvironment of endometriosis. *TNFA* and *IL6* help to sustain chronic inflammation. They can also increase *VEGFA* expression and *MMP* activity. This promotes angiogenesis, remodelling of the extracellular matrix and invasion of the lesion. This functional interplay supports their combined relevance as complementary biomarkers associated with disease progression and potential treatment response [49]. Moreover, most of the analysed molecules have also been evaluated in clinical studies, showing significantly elevated expression levels in patients with endometriosis compared to healthy controls [50–53]. Although none of these biomarkers appears to be specific to endometriosis, they are all involved in processes that are essential for its development, which makes them frequent therapeutic targets when assessing the effectiveness of new treatments.

When biomarker profiles were analysed according to the type of stem cell administered, a pattern emerged reflecting the therapeutic potential of each cell type, showing that not all cell types used as therapies exert their effects on endometriosis through the same mechanisms. ADSCs are known to possess marked regenerative, immunomodulatory and anti-inflammatory capacities [54]. This behaviour was observed in both the in vivo and in vitro studies analysed in the present review. In these studies, the cells mainly reduced inflammation and inhibited fibrosis, among other effects. In recent years, these cells have attracted increasing attention due to their ease of isolation and characterisation, their immunomodulatory capacity and their low immunogenicity [55], which likely explains the widespread use of these stem cells and their derived products in many of the studies included in this review. This anti-inflammatory activity was also observed in BMMSCs and MenSCs, in line with previous studies [56, 57], with both cell types showing effects similar to those reported for ADSCs. In addition, BMMSCs led to improvements in fertility, as has likewise been reported in studies of other related conditions [58]. By contrast, the therapeutic profile of eMSCs indicates that these cells primarily affect the PI3K/AKT/mTOR pathway, which is involved in several processes essential to endometriosis, including cell growth and proliferation, apoptosis and angiogenesis [59]. Studies in other pathological contexts suggest that these cells can activate this pathway, thereby promoting angiogenesis and cell survival [60], which would be detrimental in endometriosis. However, these observations are at odds with the findings of the present review, in which these cells were reported to inhibit angiogenesis and cellular proliferation.

WJSCs were found to influence the expression of biomarkers more closely associated with apoptosis and cellular proliferation. These findings were subsequently supported by the observed effects, which included a reduction in proliferative capacity, an increase in apoptosis and decreased cell viability. This behaviour has also been described in other diseases, such as ovarian cancer [61].

In the case of some therapies, such as those based on ADSCs and UCMSCs, certain effects, for example angiogenesis in the case of ADSCs, have been reported to decrease, remain unchanged, or even increase when the same cell type is used under different experimental conditions. This apparent inconsistency may be explained by the strong context dependency of stem cell behaviour, which is influenced by the experimental model, microenvironmental cues, cell dose, route of administration and timing of evaluation [62]. In this regard, some of these limitations may be mitigated by the use of stem cell-derived products, such as conditioned media or extracellular vesicles, which reduce variability associated with cell survival, engraftment and host interaction and may provide more controlled, reproducible and stable therapeutic effects. Although some negative and neutral effects of stem cell-derived products were identified in the present work, none of these were observed in in vivo studies.

Finally, the clear predominance of positive effects over the negative and neutral outcomes identified in the present review supports the view that therapies based on stem cells and their derived products represent a promising strategy for the treatment of endometriosis. Intraperitoneal and intravenous routes were the two most commonly used methods of administration and were associated with the highest number of positive effects. These findings are consistent with other reports indicating that intravenous delivery is generally recommended for systemic or inflammation-driven disorders that may involve multiple organs, whereas intraperitoneal administration is often preferred for organ-specific conditions [63, 64]. Given that endometriosis is fundamentally an inflammatory disease with the potential to affect both pelvic and extra-pelvic organs [1], these routes appear to be the most rational choices.

This study has several limitations. Firstly, most of the studies included in the present review were conducted over a short study period, which prevents the assessment of long-term effects or the safety of the therapies used. It is also important to highlight the high risk of bias present in the studies included in this review, particularly regarding the methods of randomisation and the lack of blinding during outcome assessment, as well as potential additional biases that may have been introduced by the scoring system used for the initial risk of bias evaluation [65]. Therefore, the results of this study should be interpreted with caution. Finally, the heterogeneity in the methods of induction and characterisation of the models, as well as in the different therapies administered, impacted both the analysis of biomarkers and the assessment of therapeutic effects, thereby preventing the extraction of sufficient data to perform a more detailed analysis.

## Conclusion

This systematic review provides a comprehensive synthesis of preclinical evidence on stem cell-based therapies and their derived products in endometriosis and identifies key methodological and translational gaps in the field. Despite promising therapeutic effects, the lack of standardised experimental protocols and absence of specific biomarkers remain major barriers to clinical translation. By defining these challenges and highlighting consistent therapeutic trends, this review establishes a roadmap for future preclinical research and supports the rational development of stem cell-based strategies for endometriosis treatment.

## Supplementary Information

Supplementary Materials 1. Supplementary Figure 1. Risk of bias of the included studies.

Supplementary Materials 2. Supplementary Table 1. Data extracted from *in vitro* articles.

Supplementary Materials 3. Supplementary Table 2. Data extracted from *in vivo* articles.

## Data Availability

The datasets obtained during the present review are available from the corresponding author upon reasonable request.

## Abbreviations

ADSCs: Adipose-derived stem cells
BMMSCs: Bone marrow mesenchymal stem cells
COX-2: Cyclooxygenase-2
ELISA: Enzyme-linked Immunosorbent Assay
eMSCs: Endometrial mesenchymal stem cells
ERβ: Oestrogen receptor beta
GnRH: Gonadotrophin-releasing hormone
HUVEC: Human umbilical vein endothelial cells
IF: Immunofluorescence
IHC: Immunohistochemistry
MenSCs: Menstrual blood-derived mesenchymal stem cells
*MMPs*: Metalloproteinases
NO: Nitric oxide
N.S.: Number of studies
NSAIDs: Non-steroidal anti-inflammatory drugs
PCR: Polymerase Chain Reaction
PRISMA: Preferred Reporting Items for Systematic Reviews and Meta-Analyses
TAC: Total antioxidant capacity
TUNEL: Terminal deoxynucleotidyl transferase-mediated dUTP nick end labelling
UCMSCs: Umbilical cord mesenchymal stem cells
WB: Western blotting
WJSCs: Wharton's jelly stem cells
