# Supplementary material for: Stem cell therapy-based approaches in experimental endometriosis: a systematic review
Source: Reprod Biol Endocrinol. 2026 Apr 17;24:54. doi: 10.1186/s12958-026-01553-w (PMC13218035; doi:10.1186/s12958-026-01553-w)
Supplement: Supplementary file 1 — Supplementary Materials 1. Supplementary Figure 1. Risk of bias of the included studies. [file 12958_2026_1553_MOESM1_ESM.docx]

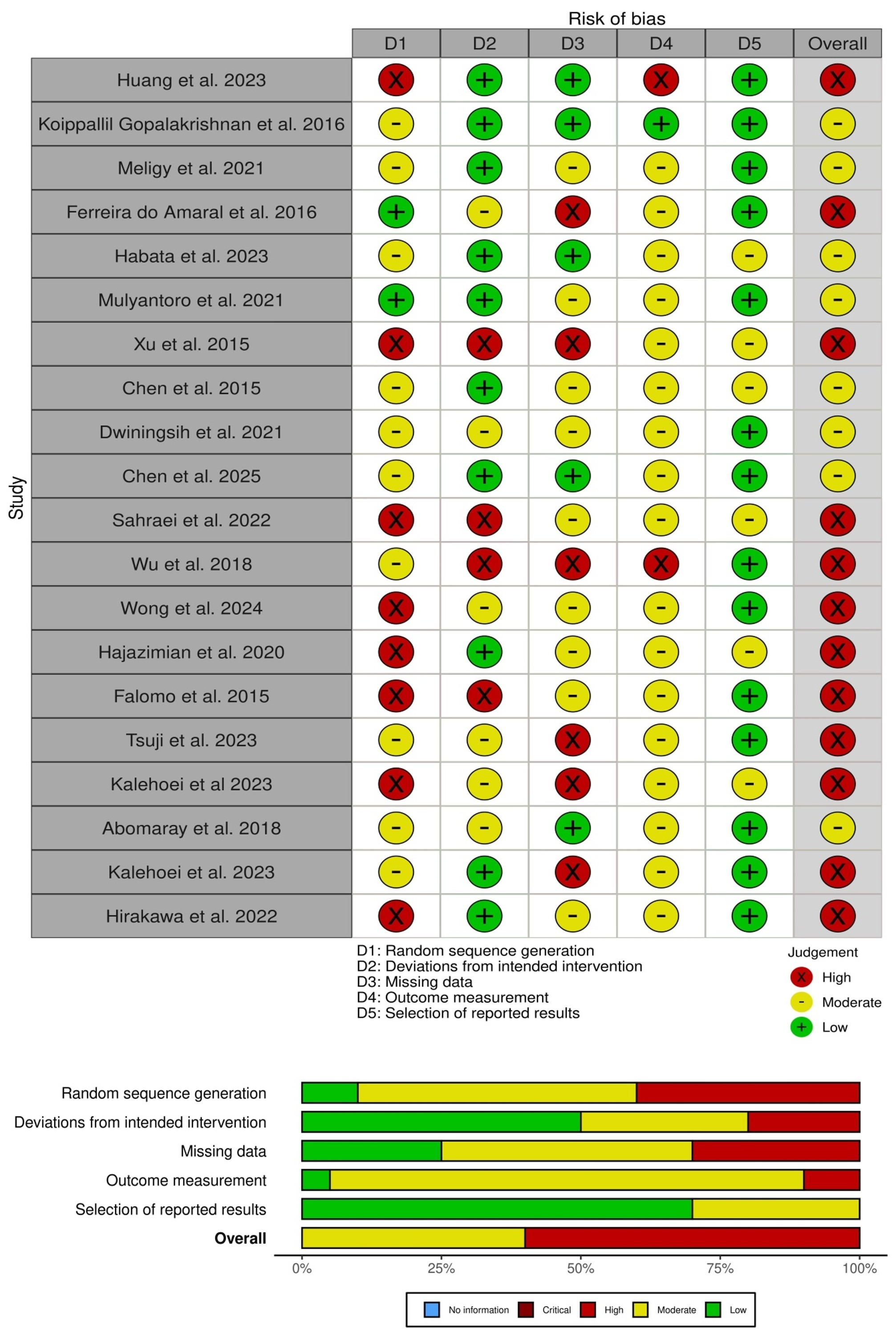


**Supplementary Figure 1:** Risk of bias assessment of the included studies generated by the robvis tool.
